# Solid-Supported Polymer Membranes: How Different Deposition Methods Influence Their Inner Morphology and Properties

**DOI:** 10.1021/acs.langmuir.5c00105

**Published:** 2025-04-23

**Authors:** Moritz S. Muthwill, Manuel Kraus, Maryame Bina, Mirela Malekovic, Ionel Adrian Dinu, Cornelia G. Palivan

**Affiliations:** † Biointerfacing Nanomaterials Group, Department of Chemistry, University of Basel, Mattenstrasse 22, BPR 1096, Basel 4002, Switzerland; ‡ NCCR Molecular Systems Engineering, Mattenstrasse 22, BPR 1095, Basel 4002, Switzerland

## Abstract

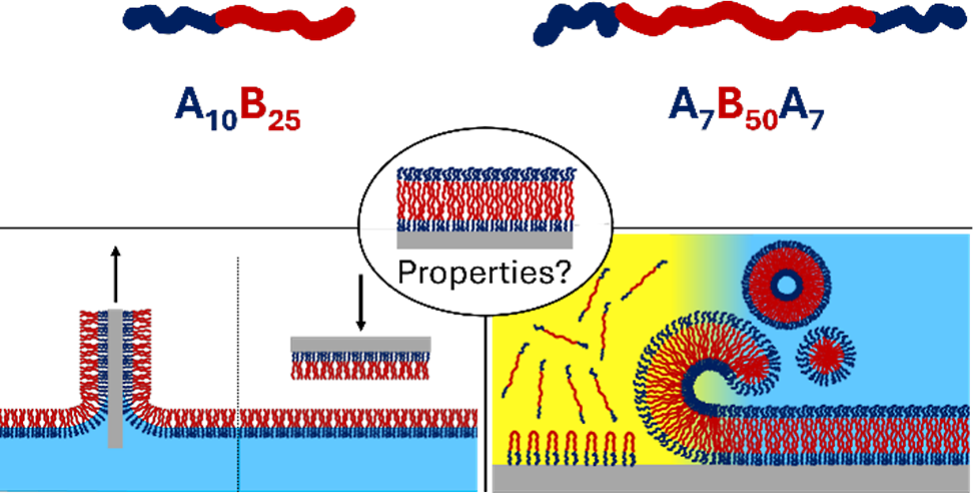

Solid-supported polymer membranes (SSPMs) resulting from the self-assembly of amphiphilic block copolymers are in focus for the development of functional surfaces with enhanced stability and more chemical versatility compared to lipid-based membranes. Different methods are used to generate such polymer planar membranes, but their internal organization and the resulting properties have not yet been compared to identify the differences and guide the selection of appropriate membranes for specific applications. Here, we present SSPMs prepared by two deposition methods—the Langmuir monolayer transfer and the solvent-assisted polymer deposition—and evaluate their internal organization and key properties. Using two different amphiphilic copolymers, a diblock and a triblock, we explored the effect of the deposition method on the resulting membranes. While the Langmuir monolayer transfer method generates condensed, homogeneous planar membranes, the ones produced by the solvent-assisted polymer deposition method have a different molecular organization. Properties, including thickness, wettability, roughness and elasticity were influenced by the membrane deposition method. Differences in inner morphology and properties of membranes generated by these two deposition methods are more pronounced for triblock-based copolymer membranes than for diblock copolymer ones. Our comparative study highlights the importance of selecting a specific preparation method to achieve SSPMs with properties tailored for desired applications.

## Introduction

Solid-supported polymer membranes (SSPMs) have emerged as promising platforms in materials and life sciences due to their chemical versatility, enhanced mechanical properties, and superior stability compared to lipid membranes. Amphiphilic block copolymers (BCPs) are particularly appealing to generate planar membranes by self-assembly,^1^ because such membranes are sufficiently soft to allow the insertion of sensitive molecules, such as membrane proteins^2^ or catalytic nanocapsules,^3^ without compromising their functionality. In addition, they can be functionalized by attaching a variety of molecules (e.g., DNA,^4^ antimicrobial peptides^5^) or nanoassemblies^3^ to gain specific properties. SSPMs composed of one type of amphiphilic BCP^3^ or mixtures of copolymers^6^ have been successfully combined with molecules and nanoassemblies. This demonstrates their high potential for developing functional surfaces in applications including catalysis,^7^ medicine,^8^ or degradation of pollutants.^9^

While the preparation methods of polymer-based planar membranes are in principle analogous to those used for lipid membranes, the conditions are significantly different and continuous optimization is essential for improving membrane properties and functionality. Typically, SSPMs are prepared by Langmuir monolayer transfer methods; however, alternative approaches, such as vesicle fusion and solvent-assisted deposition have also been applied. The Langmuir monolayer transfer methods^10,11^ are based on self-assembly of amphiphilic BCPs at the air–water interface, with deposition achieved through either vertical dipping (Langmuir–Blodgett, LB) or horizontal dipping (Langmuir–Schaefer, LS), depending on the orientation of the solid substrate relative to the water surface.^12−15^ These methods offer precise control over surface pressure and transfer speed, enabling the formation of homogeneous, defect-free membranes by complete monolayer transfer. However, extensive cleaning of the deposition equipment with harmful solvents, such as chloroform, hexane, benzene, or methanol is required.^16^ In addition, a dust-free environment is necessary to maintain a contaminant-free water surface and to ensure the integrity of the deposited film. Therefore, the solvent-assisted polymer deposition method (SAPD) was developed as an alternative to the LB/LS deposition for SSPM formation.^3,7,17^ This approach is based on the gradual solvent exchange from a water-miscible organic solvent to an aqueous buffer that induces transient self-assembly of amphiphilic BCPs into vesicles and micelles, which then fuse into a planar membrane in the presence of a solid support. Compared to LB/LS methods, SAPD offers practical advantages, as it is based on a less time-consuming one-step membrane formation process in a flow cell with less cleaning and reduced exposure to hazardous chemicals.^18^ In addition, SSMPs can be directly formed on sensors, such as those of a quartz crystal microbalance with dissipation monitoring (QCM-D) setup. This facilitates subsequent in situ modifications of deposited membranes and real-time assessments of their interactions with various molecules of interest.

Both LB/LS and SADP methods result in formation of SSPMs, but they operate under completely different conditions. LB/LS transfer methods involve the ex situ monolayer assembly at the air–water interface before the deposition on a solid support, while the SAPD method comprises the in situ self-assembly of amphiphilic BCPs on the solid support. These differences in membrane formation process, associated with the specificity of the deposition methods, are expected to induce changes in the internal organization and properties of the resulting SSPMs. They limit the understanding and selection of the most appropriate method when SSPMs with specific properties are desired or when comparisons with other reported examples are necessary. However, to the best of our knowledge, SSPMs developed by these methods have not yet been investigated and compared together.

**Figure 1:**
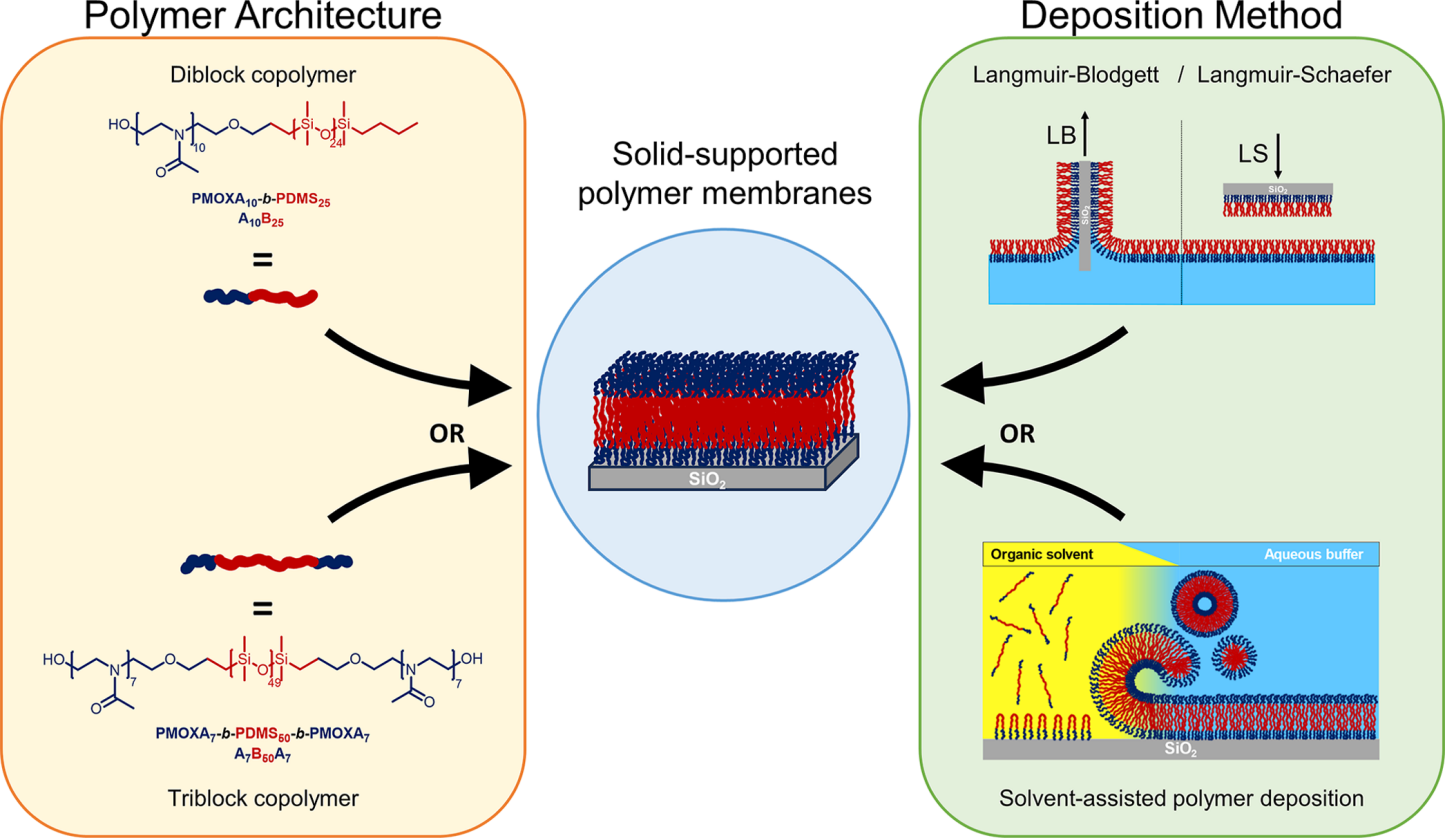
Schematic representation of two amphiphilic block copolymers with distinct block architectures and the LB/LS and SAPD methods for solid-supported polymer membranes. The illustrations depict the chemical structures and the membrane formation steps, which were investigated in all combinations.

We cover this gap by developing SSPMs based on the same amphiphilic block copolymers by LB/LS and SAPD methods, respectively, and compare their inner morphology and resulting properties. We selected two amphiphilic BCPs based on poly(2-methyl-2-oxazoline) (PMOXA) and poly(dimethylsiloxane) (PDMS) and explored how the preparation method affected the resulting planar membranes. We investigated an AB diblock copolymer, PMOXA_10_-*b*-PDMS_25_ (referred to as A_10_B_25_), and a symmetrical ABA triblock copolymer, PMOXA_7_-*b*-PDMS_50_-*b*-PMOXA_7_ (denoted as A_7_B_50_A_7_) (Figure 1). The selected BCPs, with their architecture and block chemistry, serve as ideal models for biomimetic membrane structures: PMOXA is known for its biocompatibility and nonfouling properties.^8^ On the other hand, PDMS offers flexibility due to its low glass transition temperature, facilitating functional insertion of membrane proteins, peptides, and ionophores.^19−22^ The different block architectures of the diblock and triblock copolymers are expected to provide insights into the advantages and potential limitations of each deposition method.

First, SSPMs were generated using the LB/LS and SAPD methods under conditions optimized to be as comparable as possible without affecting the deposition process. For LB/LS, the phase behavior of copolymers, which is critical for membrane deposition, was assessed at the air–water interface by recording surface pressure–area isotherms.^23^ The SAPD conditions were adapted based on previous reports describing SSPMs with PDMS and PMOXA domains.^3,7^ Second, the resulting membranes were characterized by a combination of methods to evaluate the differences in internal organization and associated properties. We measured and compared the membrane thickness by ellipsometry, the wettability by static water contact angle (CA), and the surface coverage using a bovine serum albumin (BSA) absorption assay. In addition, the membrane topography and roughness was assessed by atomic force microscopy (AFM), while the mechanical properties (elastic modulus) were investigated by force spectroscopy. The overall comparison of the SSPMs indicates effects of various strengths on the properties, that are related to the method of preparation.

The approach to generate membranes based on amphiphilic BCPs with similar chemical compositions has the unique advantage to distinguish how the preparation method should be selected when specific properties of the resulting membrane are desired. Our findings provide a deep insight into the similarities and differences in the properties of the resulting membranes, thus emphasizing the advantages and limitations inherent to each deposition method. This approach will pave the road for more efficient applications in functional coatings for sensing, nanocatalysis, water purification, or biomimetic membrane systems.

## Experimental Section

### Materials

Poly(2-methyl-2-oxazoline)_10_-*block*-poly(dimethylsiloxane)_25_ (PMOXA_10_-*b*-PDMS_25_, abbreviated as A_10_B_25_) diblock copolymer and poly(2-methyl-2-oxazoline)_7_-*block*-poly(dimethylsiloxane)_50_-*block*-poly(2-methyl-2-oxazoline)_7_ (PMOXA_7_-*b*-PDMS_50_-*b*-PMOXA_7_, abbreviated as A_7_B_50_A_7_) triblock copolymer were previously synthesized according to published protocols.^24,25^

The following chemicals were purchased from Merck KGaA: Trizma base (Tris base), sodium chloride, sodium dodecyl sulfate (SDS), bovine serum albumin (BSA, Fraction V), Milli-Q water (doubly deionized ultrapure water, resistivity ρ = 18.2 MΩ cm), and ethanol (EtOH, gradient grade for liquid chromatography, LiChrosolv). Chloroform (analytical reagent grade, 99.9% purity) was purchased from JT Baker/Avantor.

Silicon wafers (composition N/Phos, type of orientation ⟨100⟩) were obtained from Si-Mat Silicon Materials, while silicon dioxide quartz crystal microbalance sensors (QSense, model QSX 303, 5 MHz) were purchased from Biolin Scientific.

### Methods

#### Surface Pressure–Area Isotherms

Surface pressure–area isotherms were recorded using a G2 Teflon microtrough (Kibron Inc.) according to a previously established protocol.^6^ Briefly, two hydrophilic barriers were used for symmetrical film compression, and a flame-cleaned platinum Wilhelmy plate (perimeter: 8 mm) was used to monitor surface pressure (SP) with an accuracy of ±0.1 mN m^–1^. Before each measurement, the trough and barriers were thoroughly cleaned with CHCl_3_, and the trough was filled with Milli-Q water as the subphase. Any residual contaminants on the water surface were removed using a glass Pasteur pipet connected to a vacuum pump, ensuring an SP increase of ≤0.1 mN m^–1^ upon full compression. The BCPs were freshly dissolved in CHCl_3_ at a concentration of 1 g L^–1^ and slowly spread dropwise onto the water surface. The mean molecular area (MMA) (defined as the number of molecules per unit area) was calculated using the LayerX Pro/FilmWareX software based on the added solution volume, polymer concentration, and trough area (starting at 16,480 mm^2^). The polymer layer on the surface was equilibrated for 15 min at room temperature before starting compression at a barrier speed of 10 mm min^–1^. The surface pressure–area isotherms were recorded, and the collapse point (maximum SP) was determined from the average of the maximum SP values of at least three measurements.

#### Langmuir Monolayer Transfer (Langmuir–Blodgett/Langmuir–Schaefer Deposition)

Langmuir monolayers were deposited onto silicon wafer pieces (2 × 2 cm) using the Langmuir–Blodgett (LB) and Langmuir–Schaefer (LS) methods as previously described.^6,23,26,27^ In brief, silicon substrates were cleaned with EtOH and Milli-Q water and dried with compressed air. The cleaned wafers were subsequently rendered hydrophilic by UV/O_3_ treatment (Uvo Cleaner, Model No. 42A-220, Jelight Company Inc.) for 20 min. An identical setup to the one used for the Langmuir isotherms but equipped with a dipping module was used for membrane deposition. Before forming the polymer monolayer at the air–water interface, the wafers were submerged in the water subphase, and the polymer monolayers were prepared as described for the surface pressure–area isotherms. Membranes composed of triblock copolymers (A_7_B_50_A_7_) were transferred through a single LB step, thus yielding a monolayer. In contrast, diblock copolymer (A_10_B_25_) membranes were prepared by sequential LB (upstroke) and LS (downstroke) deposition steps to form a bilayer. The deposition was performed at a constant SP of 41.5 mN m^–1^ (∼80% of the collapse point). The LB upstroke velocity was set to 0.5 mm min^–1^. LS deposition was performed at a downstroke speed of 10 mm min^–1^. After contact with the water surface, the sample was equilibrated for 1 min and retracted at 0.5 mm min^–1^. The transfer ratio (TR) was calculated using the following formula (eq 1):^15^
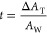
1where Δ*A*_T_ is the area change of the trough during deposition (see Figure 3), and *A*_W_ is the area of the immersed wafer. To account for the background loss of material, Δ*A*_T_ was calculated as
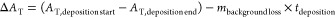
2where *m*_background loss_ is the slope of the SP baseline determined by linear regression over a time range of 10 min, when the polymer is compressed at the selected SP in equilibrated state before deposition. Values from three replicates (*N* = 3) were used for each sample.

After deposition on silicon wafers, both membranes (monolayered and bilayered) were characterized by ellipsometry (thickness in air), static contact angle (CA, wettability), and AFM (topography and mechanical properties in water). The surface coverage was evaluated using a BSA adsorption assay performed in a QCM-D setup. For this purpose, the membranes were deposited on a SiO_2_-functionalized QCM-D sensor rather than on a pure silicon wafer before insertion into the QCM-D flow cell. The membranes were thoroughly rinsed with Milli-Q water at a low flow rate (20 μL min^–1^ for 1 h, then 100 μL min^–1^ for 10 min) to wash off the residual polymers and then incubated, if necessary, with Tris-buffered saline (TBS) (100 μL min^–1^ for 10 min).

#### Quartz-Crystal Microbalance with Dissipation Monitoring (QCM-D)

QCM-D experiments were conducted as previously described.^3^ Briefly, up to 4 simultaneous measurements were performed using a QSense Analyzer together with QSoft 401 (v. 2.8.5) as control software (Biolin Scientific) and a 4-channel Reglo Digital peristaltic pump (Ismatec). Tygon MHSL tubing with two stoppers was used for the peristaltic pump and then connected to PTFE tubing (ID: 0.75 mm). Values of the resonance frequencies and dissipation of the fundamental harmonic and odd-numbered overtones (3rd to 13th) were recorded at room temperature (23.4 °C). Analysis of frequency and dissipation, thickness determination, and data fitting were performed using the Dfind software (v. 1.2.8, Biolin Scientific).

#### Solvent-Assisted Polymer Deposition (SAPD)

SAPD was conducted in a QCM-D flow cell using SiO_2_-functionalized QCM-D sensors (denoted as QCM-D sensors) or laser-cut, round silicon wafers (14 mm diameter). Both exhibit similar surface chemistry after activation and are suitable for membrane formation (Figure S1). When the deposition was performed on a round-cut silicon wafer, another deposition was conducted in parallel on a QCM-D sensor as internal deposition quality control. The subsequent characterizations (ellipsometry, CA, AFM) were preferentially performed on the membranes deposited on round-cut Si wafers since their roughness is lower than those of QCM-D sensors (nominal roughness < 2 nm), which ensured better comparability with the membranes deposited by LB/LS techniques. QCM-D sensors and silicon wafers were first cleaned by soaking in 2% SDS solution for 30 min, rinsing with Milli-Q water and EtOH, drying with N_2_, UV-Ozone cleaning for 20 min, rinsing again with Milli-Q water, and then drying with N_2_. Before membrane deposition, the substrates were placed into the QCM-D flow cell. SAPD was conducted as described previously.^3,28^ For structural comparisons of the membranes, Milli-Q water was used as the aqueous phase for solvent exchange (samples referred to as SAPD_H_2_O_). Before use, Milli-Q was filtered through a 0.2 μm PVDF membrane and degassed by sonication in a water bath for at least 15 min. Stock solutions of BCPs (0.5 g L^–1^) were prepared freshly in filtered EtOH. As SAPD is frequently performed with buffer solutions, the effect of using TBS as the aqueous phase on the membrane properties was also investigated (samples referred to as SAPD_TBS_). TBS was prepared with Tris base (10 mM) and NaCl (150 mM), adjusted to pH 7.5 with HCl, and stored in the fridge at 4 °C. Prior to use, TBS was filtered and degassed following the same procedure as for Milli-Q water, but with an additional step of equilibration at room temperature for 1 h.

The SAPD protocol consisted of sequential injection steps at a flow rate of 100 μL min^–1^ for 10 min each, unless noted otherwise. The frequency (Δ*f*) and dissipation (Δ*D*) of the QCM-D flow cell in the aqueous phase were equilibrated until Δ*f* variations were less than 0.2 Hz over 10 min (step 1). The chamber was then rinsed with EtOH (step 2), followed by the injection of the polymer solution in EtOH (step 3). The solvent exchange was then achieved by gradually replacing the polymer solution with the aqueous phase (step 4). For structural characterization, the SA-deposited membranes were rinsed with Milli-Q water, removed from the flow cell, and then evaluated by ellipsometry and CA measurements. Alternatively, they were directly used in surface coverage measurements using a BSA adsorption assay. For this, BSA (5.00 g L^–1^ in TBS) was injected into flow cells containing membranes and then rinsed with TBS. The frequency shifts were recorded, and the surface coverage was calculated with eq 3, which compares the observed BSA-induced QCM-D frequency shifts of the membrane samples with those of the controls. The frequency shifts are correlated with the adsorption of BSA onto the surface (irrespective of mono- or multilayers formation), enabling the detection of membrane defects in the case of solid-supported membranes. Furthermore, all experimental conditions, including buffer and salt concentration, temperature, BSA concentration, as well as the injection and washing steps, were kept identical in all measurements. As a control, a bare substrate was used.
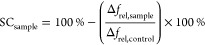
3

#### Data Analysis and Calculations

All overtones were considered for the evaluation of data quality. The reference period for Δ*f* and Δ*D* was set to 1 min at the end of the buffer baseline (step 1). For further analysis, the seventh overtone was used, as previously reported.^7,28,29^ The Δ*f* and Δ*D* values of each step were averaged over the last minute of each step. Values from *N* ≥ 3 replicates were used for each sample for calculation of the frequency and dissipation shifts after step 4. Membrane thickness was calculated using the composite Sauerbrey model, including all measured overtones (3rd–13th), considering the average at the end of step 4 (1 min), and assuming a layer density of 965 g L^–1^ (density of PDMS) for all membranes.^30^ The density and viscosity values for the aqueous phases at 23.4 °C were set as 1005 g L^–1^ and 0.92 mPa s for TBS, and 997 g L^–1^ and 0.92 mPa s for Milli-Q water.^3^

The ratio of dissipation and frequency (Δ*D*/(−Δ*f*)) was calculated with Δ*D* and Δ*f* values of step 4. The relative frequency shifts of Δ*f* were calculated for all samples and controls using eq 4:
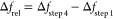
4

To estimate the ratio of frequency shifts induced by the deposition of diblock and triblock copolymer membranes, the following calculation was used (eq 5):
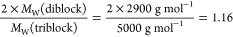
5where *M*_W_ (diblock/triblock) is the number-averaged molecular weight of the diblock or triblock copolymer, respectively.

#### Ellipsometry

Film thicknesses in air were measured using a Nanofilm EP3SW imaging ellipsometer (Park Systems) with a Nd/YAG laser (658 nm) and the EP3 view V235 software according to the manufacturer’s nulling ellipsometry protocol (AOI variation_four zone). Specific starting settings were: laser power: 2%, angle of incidence (AOI): 50°, polarizer: 50°, compensator: 45°, analyzer: 30°. The AOI was varied from 50° to 55° with an increment of 1°. The delta and psi values were transferred to the EP4 software model and fitted to an optical model using a n_k_fix dispersion term with a refractive index of 1.5 for the polymer layer. Three spots were measured for each sample, calculating their mean value and the corresponding standard deviation. To estimate the homogeneity of the polymer films, microscopic contrast images were recorded at an AOI of 50° for each region (Figure S2).

#### Static Water Contact Angle

The static water contact angle of SSPMs was measured using the sessile drop method with a Drop Shape Analyzer DSA25 (Krüss). A 2 μL droplet of Milli-Q water was placed onto the membrane surface with a microsyringe, while the process was recorded with a CCD camera (IDS, Germany). After a droplet stabilization period of 10 s, the CA was calculated as the average of 10 measurements taken over 10 s using the software of the instrument (Krüss ADVANCE v.1.7.2.1). The final CA value was determined as the average CA from at least three different spots on each sample.

#### Atomic Force Microscopy (AFM) and Force Spectroscopy

Topographical imaging and force spectroscopy measurements were performed using a NanoWizard 3 AFM (JPK Bruker) and the SPM control software. The surface topography of the polymer membranes was investigated in intermittent contact (tapping) mode with the samples immersed in Milli-Q water at room temperature. Commercially available SCANASYST-FLUID cantilevers (Bruker) were used for imaging, with a nominal resonant frequency of 150 kHz and a nominal spring constant of 0.7 N m^–1^. Images were analyzed using the JPK SPM Data Processing software (v. 8.1.8).

Force spectroscopy measurements were conducted in force mapping mode using Biosphere B20-FM probes (nanotools GmbH) with spherical tips of defined radii (20 ± 5 nm, individually determined by the manufacturer). The probes had a nominal resonance frequency of 75 kHz and a nominal spring constant of 2.8 N/m. The cantilever sensitivity was calibrated prior to the measurement by acquisition of force curves on a clean silicon substrate in water, followed by calibration of the spring constant using the thermal noise method.^31^ Force maps were recorded on 10 μm × 10 μm areas with 8 × 8 pixels, measuring one force–distance curve per pixel. The force–distance curves were acquired at a constant *Z*-speed of 0.5 μm s^–1^, a contact time of 0 s, a *Z*-length of 200 nm, and a set point force of 5 nN. The elastic modulus (Young’s modulus, *E*) was determined using eq 6:
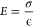
6where σ is the stress applied to the material (force per unit of cross-sectional area) and ϵ is the strain (the relative deformation defined as the ratio between the change in length of the material and its initial length).^32^ The Hertz model was used to fit the force–distance curves in the JPK SPM Data Processing software (v. 8.1.8). For the calculation of *E*, the spherical tip radii provided by the manufacturer were used, and the Poisson ratio was set to 0.5, as previously reported for membranes based on self-assembled amphiphilic block copolymers.^33^

## Results and Discussion

For comparison of the membrane properties resulting from LB/LS and SAPD methods, we selected an amphiphilic diblock and an amphiphilic triblock copolymer, both composed of hydrophilic PMOXA (A) and hydrophobic PDMS (B) blocks. With the aim to obtain comparable thicknesses of the hydrophobic domains in the resulting polymer membranes, assuming that the polymer chains adopt a completely extended conformation, we specifically chose the diblock (A_10_B_25_) and the triblock (A_7_B_50_A_7_) copolymers. Their selection ensures that the hydrophobic PDMS block in the triblock copolymer is twice the length of that in the diblock copolymer. Similarly, the hydrophilic PMOXA blocks were selected to be nearly identical in numbers of monomer units such to create membranes with hydrophilic domains of similar thickness. Both block copolymers have previously been used to successfully prepare planar membranes as well as polymeric vesicles, demonstrating their versatility in various applications.^3,6,24^ Although shorter block sequences can also induce self-assembly into membranes, they are expected to result in thinner, more fluid structures with reduced stability, which will impact the overall behavior and limit their possible applications. *f* values of about 30% (diblock) and 25% (triblock) are both in the range where planar membranes can be formed. In addition, the lengths of the polymer blocks ensure the formation of robust membranes with properties suitable to accommodate membrane proteins similar to biological membranes.^19^ In addition, in the case of the symmetric triblock copolymer, two potential chain conformations were considered to coexist within the membrane: a stretched “I-shape” and a curved “U-shape” form.^19^ Additionally, the variation in molecular weight dispersity (*D̵*), ranging from 1.19 for the diblock copolymer^34^ to 1.76 for the triblock copolymer,^3^ is also expected to affect membrane properties. Therefore, to understand in detail the structural morphology of di- and triblock copolymer SSPMs, we explored the influence of the preparation methods on the molecular organization of polymer chains within the self-assembled membranes.

## Langmuir Monolayer Transfer vs Solvent-Assisted Polymer Deposition

### Langmuir Monolayer Transfer

**Figure 2:**
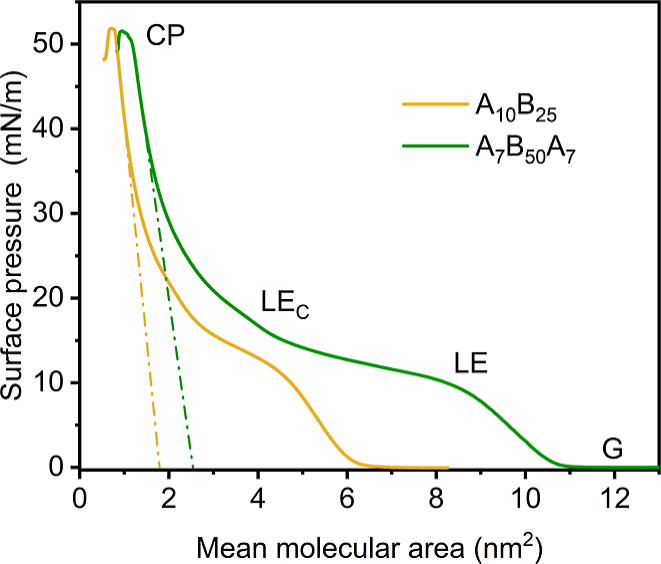
Surface pressure–area isotherms at the air–water interface for Langmuir monolayers prepared from diblock (A_10_B_25_) and triblock (A_7_B_50_A_7_) copolymers. The monolayer phases are labeled at their respective surface pressures as follows: G = gaseous, LE = liquid expanded, LE_C_ = more condensed liquid expanded, and CP = collapse point. Dashed lines represent the linear extrapolation used to determine the limiting molecular area.

Langmuir methods rely on forming a stable monolayer of amphiphilic molecules at the air–water interface, which is then transferred onto a solid support. To precisely control film formation and ensure a successful Langmuir monolayer transfer, a thorough understanding of the phase behavior of the amphiphilic block copolymers is essential. Therefore, we measured the surface pressure–area isotherms of A_10_B_25_ and A_7_B_50_A_7_ block copolymers, respectively, on water as the subphase (Figure 2). The curves displayed typical characteristics of copolymers with flexible chains composed of PDMS and PMOXA blocks.^6,35^ The main isotherm parameters for both copolymers indicated their monolayer film architecture (Table S1).

After spreading on the water surface at zero surface pressure, the individual macromolecules become hydrated, separated from each other, with their polymer chains arranged randomly at the air–water interface. PMOXA blocks, being much shorter and highly hydrophilic, orient toward the water subphase and adopt an extended coil conformation. PDMS chains, which are longer, highly flexible, and characterized by low intermolecular forces between methyl groups and a partially ionic Si–O bond, exhibit unique structural properties, including a low glass transition temperature of −123 °C and low surface activity at the air–water interface.^36,37^ Therefore, PDMS polymer chains remain uncoiled and relaxed at low surface concentrations, with a two-dimensional “flat” or “pancake” conformation, where all silicon and oxygen atoms are immersed in the water subphase.^38^

When the surface concentration of PDMS increased and the monolayer was forming, the surface pressure rose to a lift-off area of 6.11 nm^2^ per macromolecule (0.24 nm^2^ per PDMS monomer unit) for the diblock copolymer and 10.77 nm^2^ per macromolecule (0.22 nm^2^ per PDMS monomer unit) for the triblock one (Table S1). This indicates that silicon and oxygen atoms are gradually pushed out from the interface. Beyond the lift-off point, a liquid PDMS monolayer phase emerges as the individual “pancake” PDMS conformations interact.^35^ This difference in lift-off area from the gaseous phase (G) is primarily due to the reorganization of the PDMS chains upon initial intermolecular interaction at the interface. Such polymer–polymer interaction varies according to the chain length. Further compression led to the initial “collapse” of the PDMS monolayer, corresponding to a “plateau” region in the pressure–area isotherms where the surface pressure remains nearly constant. This plateau represents the transition from a liquid extended (LE) to a more condensed liquid phase (LE_C_)^35^ and is attributed to the gradual coiling of PDMS chains into “loops” or helical conformations, or even to the formation of PDMS bilayer structures.^39,40^ The starting pressure of this transition phase was dependent on the size and flexibility of the polymer chains, beginning at approximately 14 mN m^–1^ for the shorter PDMS block (A_10_B_25_) and at 11 mN m^–1^ for the longer one (A_7_B_50_A_7_). This behavior was more pronounced in the triblock copolymer due to its longer PDMS block, which offers greater flexibility and conformational freedom at the air–water interface. The diblock copolymer exhibited a transition spanning approximately 2 nm,^2^ indicating limited PDMS packing capacity due to its smaller size. Upon more compression, a condensed liquid phase formed, characterized by a significant increase in pressure with a varying slope depending on the polymer size. This region, interpreted as a second “collapse” of either the helical monolayer or the PDMS bilayer, may involve some PDMS helices orienting themselves vertically or the formation of PDMS multilayers. At this stage, the initial freedom of the PMOXA blocks becomes increasingly restricted, leading to a continuous stretching of the solvated PMOXA chains in the water subphase and a more organized arrangement of the block copolymers. This structural change induces the PDMS block of the triblock copolymer to either bend into an inverted “U-shape” conformation, with both PMOXA blocks solvated, or to move one PMOXA block out of the water, adopting a coiled “I-shape” conformation.

At much higher compressions, the submerged PMOXA blocks extended from a coil to a brush conformation, allowing all block copolymers to be compressed to nearly the same cross-sectional area, mainly defined by the conformation and steric requirements of the hydrophobic PDMS block. At this point, both polymer monolayers reached a collapse point around 51 mN m^–1^ (Table S1), a value consistent with that observed for other copolymers composed of PDMS and PMOXA blocks.^35,39^ This behavior indicates that the polymer chains have reached their maximum packing density before monolayer collapse occurs.^40^ The diblock copolymer occupied a slightly smaller area (approximately 0.2 nm^2^ less) than the triblock one, likely due to differences in molecular weight and the coexistence of both stretched “I-shape” and curved “U-shape” conformations within the triblock copolymer membrane. The limiting molecular area, indicating the area occupied by a molecule in the most condensed state, was determined by extrapolating from the linear increase before reaching the collapse point to zero surface pressure.^41^ The values for the limiting molecular areas are 1.69 ± 0.05 nm^2^ for the diblock copolymer and 2.56 ± 0.09 nm^2^ for the triblock one (Table S1). These values are indicating that the triblock copolymer chains partially retain their “U”-conformation in the compressed state, occupying more space than the diblock copolymer chains.^42^ At high pressures, the cross-sectional areas of individual polymer chains are influenced by both the chain conformation and the size of polymer side functionalities. By comparing the mean molecular areas at the collapse point with the theoretical effective cross-sectional areas of ideally stretched polymer chains in their most common conformation (estimated at approximately 0.250 nm^2^ for PMOXA^43,44^ and 0.345 nm^2^ for PDMS^45^), it becomes evident that the chains were not fully extended. This deviation from the elongated “zigzag” arrangement is partly due to the hydrophobic mismatch between individual PDMS chains of varying lengths, resulting from the relatively high *D̵* values. To match the local environment, the PDMS chains adjust their conformation by adopting helical or multilayered structures within the monolayer, leading to larger cross-sectional areas.

**Figure 3:**
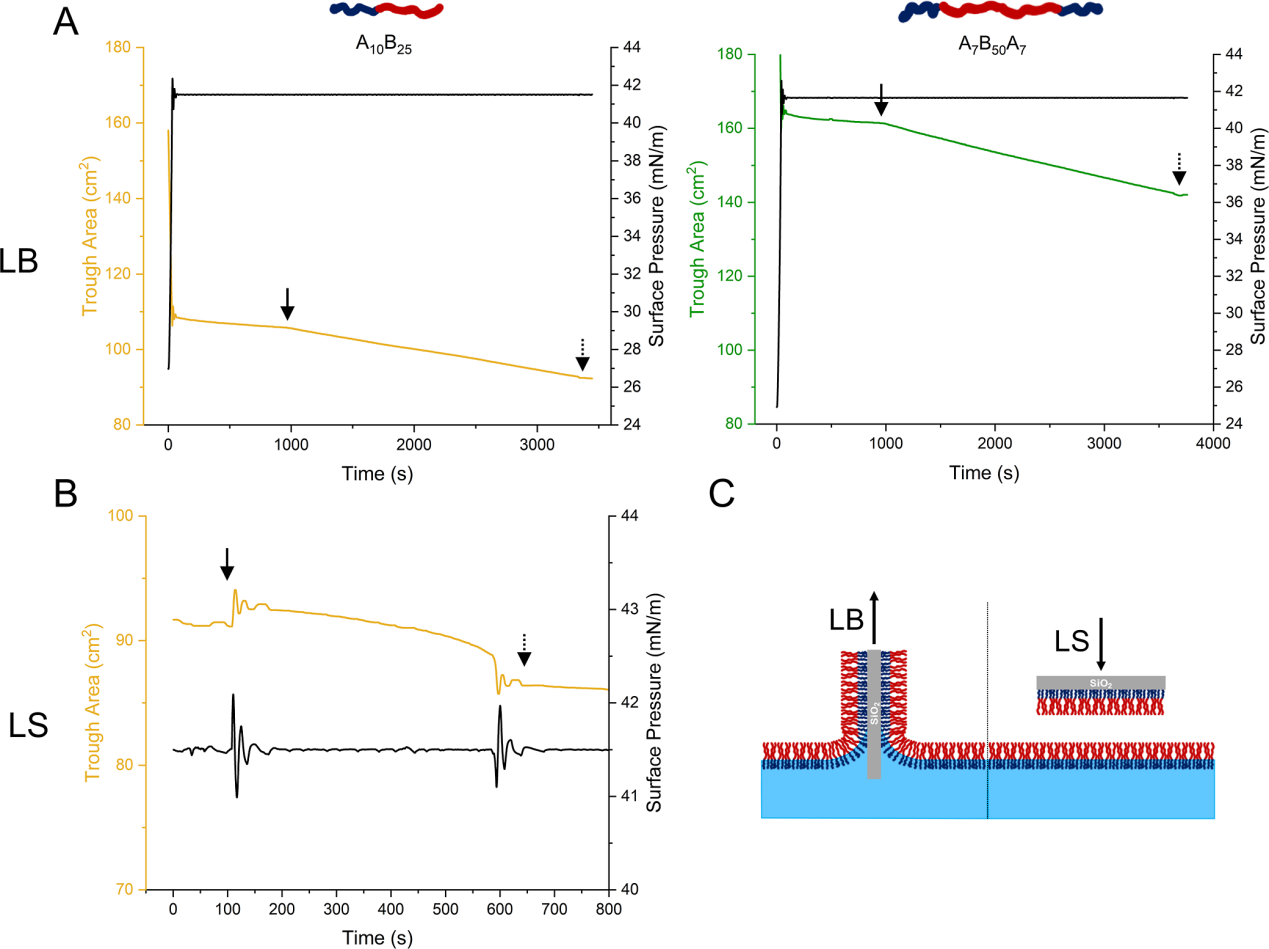
Exemplary deposition data for the diblock copolymer bilayer and the triblock copolymer monolayer on a solid support using Langmuir–Blodgett (LB) and Langmuir–Schaefer (LS) methods, respectively. The changes in trough area from the start (full arrow) to the end (dotted arrow) of each deposition were used to calculate the transfer ratios. In the LB depositions (A), two substrates were processed simultaneously, whereas the LS deposition (B) was performed on a single substrate. (C) Representative graphical depiction of both deposition steps for the diblock copolymer. For the triblock copolymer, only LB depositions were performed.

Langmuir monolayer transfers were performed at constant surface pressure within the LE_C_ phase. Polymer membranes of either diblock or triblock copolymer were deposited using sequential LB and LS steps or a single LB step, respectively (Figure 3). The transfer ratio (TR) of each deposition step was calculated using the decrease of the monolayer area between both barriers during the process (eqs 1, 2 and Table 1).

**Table 1:** Transfer Ratios Calculated for Langmuir Monolayer Transfer Methods (LB and LS) of Diblock (A_10_B_25_) and Triblock (A_7_B_50_A_7_) Copolymers^a^

polymer	layer	transfer ratio (TR)
A_10_B_25_	1 (LB)	0.92 ± 0.08
	2 (LS)	0.85 ± 0.05
A_7_B_50_A_7_	1 (LB)	1.05 ± 0.06

a Transfer ratios provide an estimate of deposition quality.

The TR values provide an estimate of the monolayer transfer efficiency from the air–water interface to the solid support, with TR values of 1 indicating complete transfer, while values below 1 suggest incomplete transfer. For the block copolymers investigated, the TR values on pure silicon wafers indicate near-complete transfer (TR ≅ 0.9) for the diblock copolymer and complete transfer (TR ≅ 1) for the triblock one. It is important to mention that the TR calculations may be slightly influenced by experimental uncertainties that are difficult to estimate, e.g. polymer deposition on wafer edges. Such small effects may contribute to the observed average TR value exceeding the theoretical limit of 1. Most notably, the linear decrease in the trough area observed prior to deposition reflects the loss of a small fraction of amphiphilic species from within the trough barriers. This background loss was accounted for in the TR calculations (Table S2). This loss was particularly pronounced for the diblock copolymer, potentially indicating a greater monolayer mobility. Additionally, during the LS step for the diblock copolymer (Figure 3B), the surface pressure and trough area showed significant fluctuations as the substrate entered and exited the water subphase, resulting in irregularities in the curve. To minimize the calculation errors in the TR for the LS transfer, trough area values of the stable periods immediately before and after deposition were considered (full/dotted arrows in Figure 3).

### Solvent-Assisted Polymer Deposition

**Figure 4:**
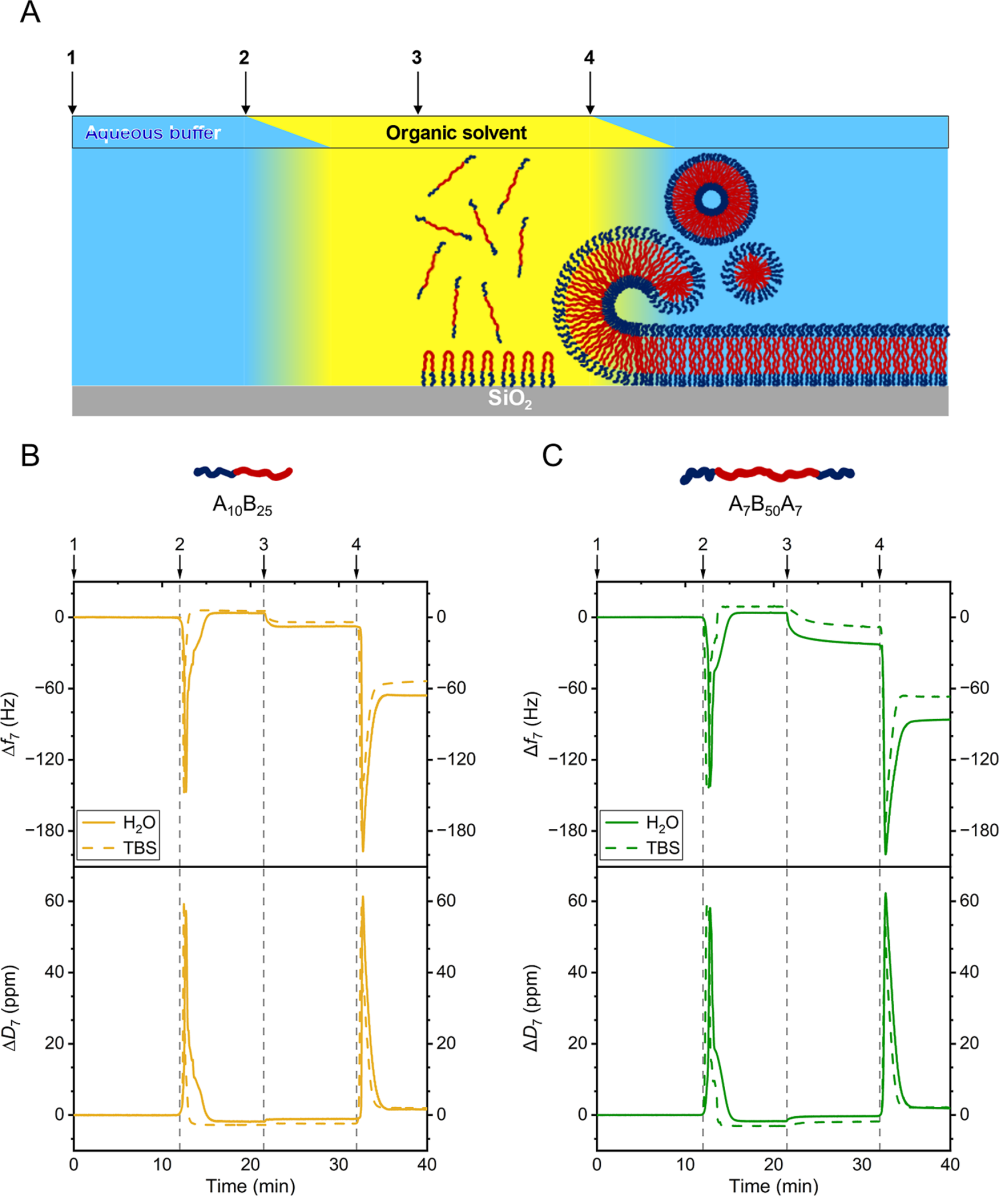
(A) Graphical representation of the solvent-assisted membrane formation process for the triblock copolymer. Injection steps are indicated by numbered arrows above the graph and dashed lines within the plot: (1) aqueous phase baseline, (2) ethanol (EtOH), (3) block copolymer solution in EtOH, and (4) aqueous phase (solvent exchange). The aqueous phase (steps 1 and 4) consisted of either H_2_O or TBS. Exemplary data from QCM-D monitoring of solvent-assisted diblock (B) and triblock (C) polymer membrane deposition on SiO_2_-functionalized sensors. Changes in frequency (Δ*f*) and dissipation (Δ*D*) were recorded over time.

In contrast to the Langmuir monolayer transfer methods, SAPD relies on the self-assembly of copolymers in the aqueous phase or directly at the solid–water interface upon solvent exchange. The process enables of membrane formation within a sealed flow cell and without the need for careful monolayer preparation. Additionally, membrane formation can be monitored in situ using QCM-D (Figure 4). The parameters for SAPD with PMOXA-*b*-PDMS-based copolymers were optimized based on established approaches.^3,7^ Typically, solvent-assisted deposition of both polymer and lipid membranes is conducted using buffers such as Tris-buffered saline (TBS) or phosphate-buffered saline (PBS) as the aqueous phase.^46^ However, to allow for a proper comparison of the resulting membranes with those obtained by Langmuir monolayer transfer methods conducted in pure water, we self-assembled the block copolymers in Milli-Q water as well, to avoid the influence of salts on the internal structure of the resulting membrane. This approach resulted in SAPD_H2O_ membranes, which were then compared with the membranes obtained by LB/LS deposition. Subsequently, TBS was used as the aqueous phase, leading to SAPD_TBS_ membranes. which were then compared with the SAPD_H_2_O_ membranes to get insight into the role of the aqueous phase composition on the internal organization of the membrane.

The SAPD process started with an initial switch from an aqueous baseline (H_2_O or TBS, Figure 4, step 1) to the water-miscible organic solvent (EtOH, step 2), clearly visible as a strong dip in frequency. This frequency dip is caused by the immediate change in density and viscosity of the bulk liquid above the sensor during the solvent exchange process. Injection of the diblock or triblock copolymer solutions in EtOH (step 3) caused a slight frequency decrease, indicating a minor attachment of polymer on the surface. After changing back to the aqueous phase (H_2_O or TBS, step 4), a frequency shift compared to the baseline confirms the deposition of material on the substrate. For the SAPD_H2O_ membranes, frequency shifts of −66 ± 8 Hz for the diblock and −75 ± 8 Hz for the triblock copolymer were recorded, corresponding to thicknesses of 12.2 ± 1.5 nm and 14.0 ± 1.4 nm, respectively, as calculated by the composite Sauerbrey model (Table 2). In the case of SAPD_TBS_ membranes, frequency shifts of −57 ± 7 Hz for the diblock and −70 ± 5 Hz for the triblock copolymer were observed, corresponding to thicknesses of 10.1 ± 1.2 nm and 13.0 ± 1.0 nm, respectively. Additionally, a small energy dissipation change (∼2 ppm) was observed for all membranes, suggesting similar overall viscoelastic properties. The Δ*D*/(−Δ*f*) ratio, with values an order of magnitude below the threshold of 4.0 × 10^–7^ Hz^–1^, indicates that all membranes can be approximated as being rigid.^47^

**Table 2:** Characteristic Data of Diblock and Triblock Copolymer Membranes Prepared by the SAPD Method^a^

aqueous phase	polymer	Δ*f* (Hz)	Δ*D* (ppm)	thickness (QCM-D) (nm)	Δ*D*/(−Δ*f*) (Hz^–1^)
H_2_O	A_10_B_25_	–66 ± 8	2.0 ± 0.2	12.2 ± 1.5	(3.1 ± 0.5) × 10^–8^
	A_7_B_50_A_7_	–75 ± 8	2.1 ± 0.3	14.0 ± 1.4	(2.9 ± 0.4) × 10^–8^
TBS	A_10_B_25_	–57 ± 7	2.2 ± 0.3	10.1 ± 1.2	(3.9 ± 0.8) × 10^–8^
	A_7_B_50_A_7_	–70 ± 5	2.1 ± 0.4	13.0 ± 1.0	(3.0 ± 0.5) × 10^–8^

a Δ*f* and Δ*D* represent shifts observed during membrane formation by SAPD, as measured by QCM-D. Thickness (QCM-D) was calculated using the composite Sauerbrey model. The Δ*D*/(−Δ*f*) ratio was used to assess the mechanical properties (rigid or viscoelastic behavior) of the polymer membrane, with values significantly less than 4.0 × 10^–7^ Hz^–1^ indicating a rigid behavior.^47^

Overall, these data demonstrate the successful formation of planar diblock and triblock copolymer membranes using SAPD in pure water and TBS, respectively. However, the presence of salts resulted in slightly thinner membranes (1–2 nm), likely due to changes in molecular organization or variations in the dynamics of the self-assembly process. This effect may also have been influenced by the “salting-out” effect, where the salts of the TBS buffer induced a conformational change in the polymer chains, promoting irregular packing and reducing membrane thickness.

The relative intensities of the observed frequency shifts and the corresponding membrane thicknesses of both block copolymers deviated from the expected intensities based on their molecular weight ratios. Assuming that a complete membrane consists of a bilayer for the diblock copolymer and a monolayer for the triblock one, a 16% higher mass was expected for the diblock bilayer compared to the triblock monolayer (for factor calculation, see eq 5). However, both SAPD_H2O_ and SAPD_TBS_ triblock copolymer membranes had higher mass and thickness compared to the diblock membranes. This difference may be due to variations in polymer conformation, such as the triblock copolymer adopting mixed “I-” and “U-shapes”,^19^ and to a higher packing density of triblock copolymer chains compared to the diblock chains in the self-assembled membrane.

## Membrane Characterization

To reveal the structural differences produced by the two different deposition methods, SSPMs were comprehensively analyzed using a combination of surface characterization techniques, including surface coverage determination, ellipsometry, contact angle measurements and atomic force microscopy.

### Surface Coverage of Planar Membranes

We determined the surface coverage of SSPMs using a bovine serum albumin (BSA) adsorption assay.^3,7,46,48^ BSA exclusively attaches to the exposed areas of the solid substrate but does not adsorb onto the copolymer membrane,^8^ resulting in a direct proportionality between the amount of adsorbed BSA and the fraction of free surface. Mass changes caused by BSA adsorption events were quantified using QCM-D after a 10 min equilibration time, ensuring that BSA adsorption reached the equilibrium, thus allowing an accurate determination of the membrane surface coverage.

The SAPD_H2O_ membranes exhibited high surface coverages (around 80%), indicating minimal membrane defects, in agreement with previous reports.^3,7^ Similarly, diblock and triblock copolymer membranes obtained by SAPD_TBS_ showed high surface coverages, reaching values above 90% (Table 3), possibly due to the reduced solubility of the polymers in buffer. On the contrary, the surface coverages of the LB/LS-deposited membranes were lower, with values around 65% for both block copolymers (Table 3), which were in contrast to the high TR values observed for pure silicon wafer substrates. A potential reason for the lower coverage of LB/LS-deposited membranes is their possible destabilization during the insertion into the liquid flow cell used for the BSA assay. The partial drying, subsequent rehydration and the mechanical contact between the sensor edge and the sealing O-ring may have contributed to the reduced surface coverage.^49^ This behavior was further confirmed by a decrease in surface coverage of up to 20% for SAPD_TBS_ membranes that were intentionally removed from the flow cell after deposition, and then rehydrated upon reinsertion (Table S3). The reduced surface coverage of the LB/LS-deposited membranes may also arise from an incomplete transfer of the monolayer onto the QCM-D sensors due to their specific topology (silicon surface in the center surrounded by a glass ring and electrodes with different surface properties). This surface topology likely interferes with the uniform monolayer transfer when the entire sensor is submerged during deposition. Note that, under optimal conditions and on pure silicon wafers, it is expected that both deposition methods generate homogeneous membranes with minimal defects over large areas.

**Table 3:** Surface Coverage of SSPMs Determined by BSA Adsorption Assay Using QCM-D^a^

block copolymer	deposition method	Δ*f*_BSA_ (Hz)	surface coverage
A_10_B_25_	LB/LS	–9.5 ± 1.0	63 ± 5%
	SAPD_H_2_0_	–5.2 ± 0.6	80 ± 3%
	SAPD_TBS_	–1.9 ± 0.8	95 ± 5%
A_7_B_50_A_7_	LB	–8.9 ± 0.6	65 ± 4%
	SAPD_H_2_0_	–5.5 ± 1.1	78 ± 4%
	SAPD_TBS_	–0.9 ± 1.0	96 ± 4%
control		–25.3 ± 2.0	0 ± 8%

a Membranes were deposited on SiO_2_-functionalized QCM-D sensors. As a control, a bare sensor was used. Values were averaged from triplicates and errors are indicated by the standard deviation.

### Planar Membrane Thickness

Thickness is a critical property of SSPMs, as it provides valuable insight into the polymer chain conformation on surfaces. We used ellipsometry to measure and compare the thickness of the solid-supported membranes, as they were deposited on a reflective substrate and exhibited high air stability.^26^ Additionally, during ellipsometry measurements, microscopic images were recorded to assess the quality and uniformity of the deposited membranes (Figure S2). Normally, membrane thickness values measured in air are expected to be lower than those for comparable polymer membranes immersed in aqueous medium (e.g., neutron reflectometry measurements of supported membranes).^3^ However, in our approach, the membranes can still be considered partially hydrated because their thickness in air was measured immediately after deposition.

**Figure 5:**
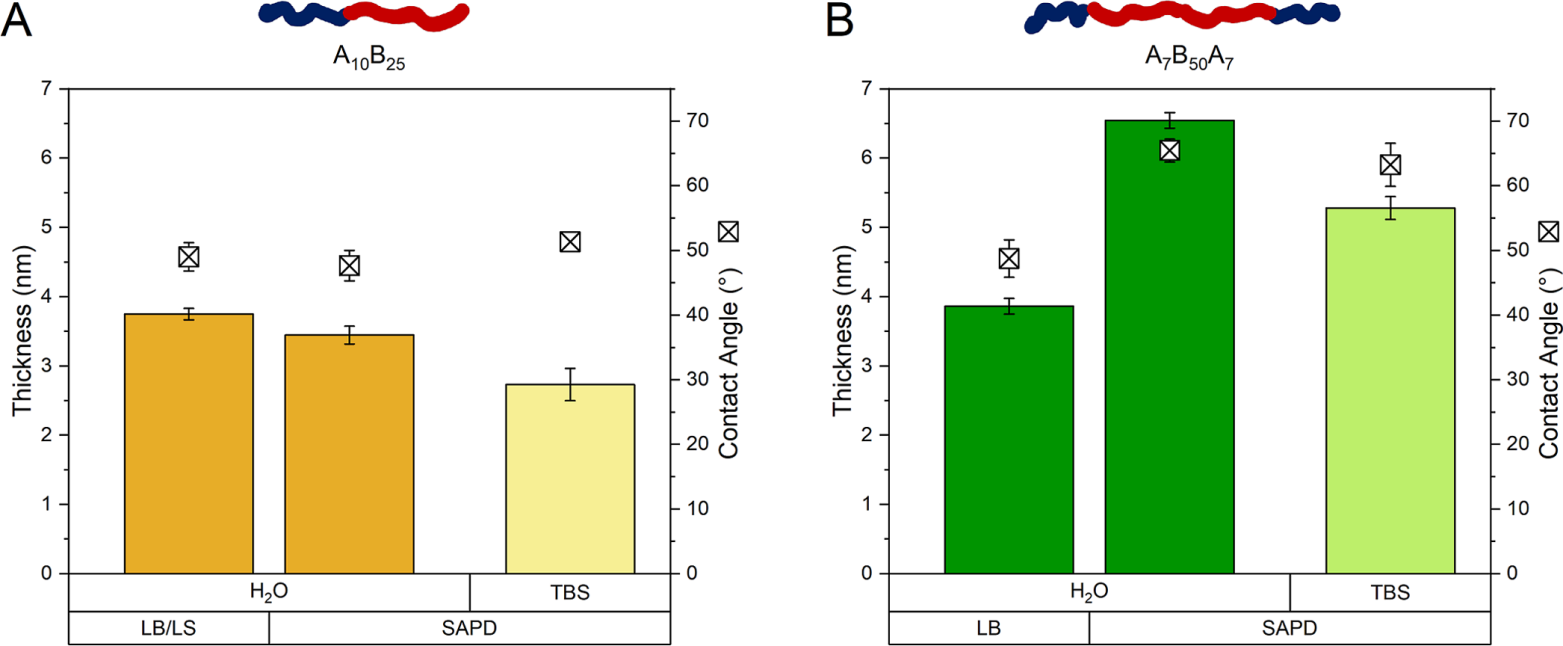
Thickness in air and wettability of diblock (A) and triblock (B) copolymer membranes prepared by LB/LS and SAPD methods as measured by ellipsometry and static water contact angle, respectively. For SAPD, results are shown for both Milli-Q water and buffer as the aqueous phase for comparison.

For membranes based on diblock copolymer deposited via the LB/LS techniques, a thickness of 3.7 ± 0.1 nm was measured (Figure 5A), slightly thinner than previously reported values for similar copolymers.^6^ Interestingly, the thickness did not double between the LB and LS steps, as would be expected after deposition of a second layer with a similar chemical composition, but only increased by about 20% (Figure S3A+B, A_10_B_25_/LB/day 0). With near-complete TRs for the LB and LS steps respectively (Table 1), an interdigitation of diblock copolymer chains through their hydrophobic PDMS blocks occurs when a bilayer is formed, in agreement with other diblock copolymer membranes.^19,50,51^ The diblock copolymer membranes obtained by the SAPD_H2O_ method exhibited a similar thickness of 3.4 ± 0.1 nm (Figure 5A) as the membranes obtained by LB/LS deposition, indicating similar inner morphology. As evidenced by the lower frequency shifts observed by QCM-D, the SAPD_TBS_ method produced slightly thinner membranes (2.7 ± 0.2 nm, Figure 5A), likely due to the “salting-out” effect of the buffer.^52^ This effect alters the self-assembly process, inducing conformational changes in the polymer chains and leading to thinner planar membranes.

In the case of triblock copolymers, LB-deposited membranes had a thickness of 3.9 ± 0.1 nm (Figure 5B), similar to the diblock bilayers produced via LB/LS deposition and consistent with the comparable total length of the PDMS blocks for both copolymers. Therefore, we consider that both diblock and triblock copolymers result in membranes with similar thickness upon transfer from the air–water interface onto the solid support, even though triblock chains are expected to adopt mixed “I-” and “U-shapes” at the air–water interface.

In contrast, triblock membranes deposited using the SAPD method in water displayed a significantly higher thickness of 6.5 ± 0.1 nm (Figure 5B), close to double of that of the diblock copolymer membranes formed under similar conditions. This indicates different layer organization of triblock copolymers between the LB and SAPD methods, with potential multilayers formation occurring during SAPD. In the SA-deposition process, the copolymers are initially noncovalently attaching to the hydrophilic silicon substrate through the adsorption of hydrophilic PMOXA blocks, as evidenced by the QCM-D frequency drop after the polymer injection (Figure 4, step 3). This initial frequency shift was approximately 10 Hz for the diblock copolymer membrane, and around 20 Hz for the triblock one, which corresponds to its roughly doubled molecular weight compared to that of the diblock. In addition, depending on the diblock or triblock nature of the copolymers, “I-shape” or “U-shape” conformations may be adopted by the copolymer chains within the adsorbed polymer membrane. During solvent exchange, polymer self-assembly lead to the formation of nanostructures such as micelles and polymersomes that rupture upon contact with the solid support.^53^ However, this rupturing process may be impaired by the polymer chains already adsorbed on the sensor surface, potentially resulting in formation of multilayers. The higher dispersity of the triblock copolymer further promotes the development of domains with various polymer chain conformations. It is therefore likely that the morphology of the triblock membrane consists of an initial layer of predominantly “U-shaped” macromolecules, and an additional layer originating from the rupture of nanoassemblies, leading to a thicker membrane.

The increased thickness of the triblock copolymer membranes generated by SAPD may also result from a different molecular packing compared to those prepared by Langmuir deposition. In the LB technique, the molecular density at the air–water interface is directly controlled by the surface pressure. In contrast, the SAPD method does not offer a direct control over monolayer formation or membrane packing. Instead, the membrane self-assembly in SAPD is influenced by parameters such as polymer concentration in the organic solvent, the rate of solvent exchange, and the choice of organic solvent. During solvent exchange, the copolymer chains are likely to adopt the thermodynamically most favored conformation in the self-assembled membrane, which may differ in packing density and chain organization compared to the membranes formed under LB conditions.

The effect of salts on the self-assembled membrane was further evident in the case of triblock copolymer SAPD_TBS_ membranes. While they exhibited a similar mechanism of formation and morphology to those formed in water, the “salting-out” effect caused the polymer chains to adopt a more coiled conformation in TBS, leading to a slightly reduced thickness of 5.2 ± 0.2 nm (Figure 5B). This decrease agrees to the lower frequency shifts observed for SAPD_TBS_ membranes in QCM-D measurements.

### Planar Membrane Wettability

To further characterize the SSPMs, we determined their surface wettability by using static water contact angle measurements. The diblock membranes exhibited similar CA values regardless the deposition methods: 49.0 ± 2.2° (LB/LS), 47.6 ± 2.4° (SAPD_H_2_O_), and 51.3 ± 1.5° (SAPD_TBS_) (Figure 5A). These values indicate hydrophilic surfaces and are in agreement with that of previously reported membranes.^6,54^ This similarity of the CA values suggests that both Langmuir methods and SAPD in water and TBS generate diblock bilayers surfaces with the upper surface layer exhibiting PMOXA copolymer block. It is important to note that freshly UV/O_3_-cleaned silicon wafers exhibit CA values close to 0°, which increased slightly over time as the surface becomes less hydrophilic (Figure S3, control, day 0 and day 7). Furthermore, the microscopic contrast images recorded during imaging ellipsometry revealed that the polymer membranes exhibit point defects (Figure S2), which were more pronounced in the LB/LS methods. These defects exposed areas of the underlying superhydrophilic silicon surface to the water droplet during CA measurements, lowering the CA values. The CA for LB-deposited triblock membranes was 48.7 ± 2.9°, comparable to the values of the diblock-based membranes, which indicates similar surface hydrophilicity. Nevertheless, the CA for SAPD triblock membranes increased to 65.5 ± 1.8° (SAPD_H_2_0_) and 63.2 ± 3.3° (SAPD_TBS_) (Figure 5B). This increase was likely due to their higher thicknesses, reducing the influence of the substrate underneath and resulting in a less hydrophilic surface.

Membrane stability of LB/LS and SAPD membranes was further assessed by measuring the thickness and CA after 7 days of storage in air (Figure S3). All membranes showed a slight decrease in thickness of less than 1 nm. This decrease is due to the dehydration of the membranes, causing the hydrophilic PMOXA blocks to adopt a more coiled conformation. However, the CA variations were dependent on the deposition method: SAPD membranes exhibited a decrease in CA, whereas Langmuir-deposited ones showed an increase. The CA changes were also more pronounced in triblock membranes than in diblock membranes, suggesting that the deposition approach and the copolymer architecture impacted the membrane stability over time, with triblock membranes being more susceptible to changes.

### Planar Membrane Topography and Mechanical Properties

**Figure 6:**
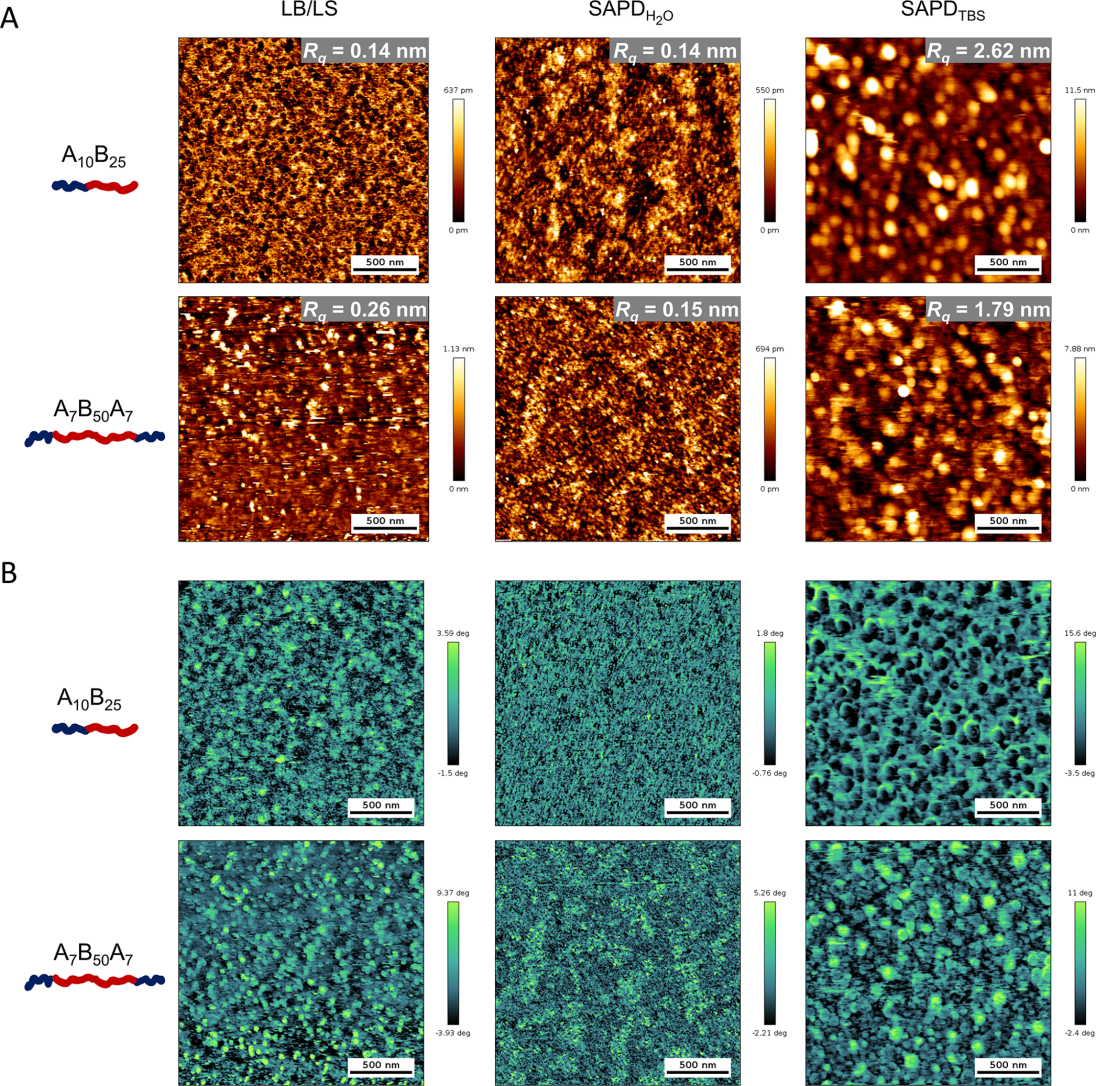
Comparison of surface morphology for diblock and triblock copolymer membranes deposited on silicon wafers using LB/LS and SAPD methods. (A) AFM height micrographs (2 × 2 μm) of membranes measured in water, with root-mean-square (RMS) roughness (*R*_q_) values indicated for each sample. (B) Corresponding AFM phase images.

Further characterization of SSPMs immediately after deposition was performed using AFM to measure their topography in water (Figure 6A). Most membranes prepared with Milli-Q water appeared flat, with maximum height differences of less than 700 pm and roughness values (*R*_q_) below 0.2 nm. However, the triblock copolymer membrane prepared by LB deposition showed a greater variation in height and roughness, possibly due to the mixture of “U-” and “I-shape” conformation arising during monolayer formation. Topography of the copolymer membranes generally varied depending on the deposition method: LB/LS-deposited membranes appeared continuous with a uniform average height, while SAPD-deposited membranes displayed irregularly shaped, bumpy domains, with darker areas indicating lower regions and lighter areas indicating higher regions (Figure 6). Evidence for these “island-like” domains can also be found in a slightly increased peak-to-valley distance for SAPD membranes, which measures the average distance from the lowest to highest point within a membrane sample (Table S4). These domains were likely the result of random pre-adsorption of a small fraction of polymer chains during the injection of the polymer solution in the SAPD process (Figure 4, step 3). On the other hand, the SAPD_TBS_ membranes showed higher roughness values of approximately 2 nm. The “salting-out” effect, as described earlier, significantly accelerated the self-assembly process, altering the polymer chain conformations and promoting irregular membrane collapse, which significantly increases the surface roughness.

To further explore surface properties, AFM phase imaging provided insights into the chemical composition and mechanical properties of the polymer membranes (Figure 6B). For the diblock copolymer membrane deposited by the LB/LS methods, the phase image closely resembled the texture of the height image. In contrast, the phase image of the SAPD_H20_ diblock membrane showed a more homogeneous texture despite the visible domains in the height image, with a phase difference of 2.6 deg compared to 5.1 deg for the LB/LS membrane. This suggests a higher surface chemical homogeneity on the SAPD_H20_ diblock membrane, consistent with its slightly lower contact angle. These small differences in phase and CA values between the diblock membranes indicate however only subtle variations in surface properties. They can be attributed to the potential interdigitation of PDMS blocks in these membranes, regardless of the deposition method, due to the greater freedom and flexibility of the PDMS chains in diblock copolymers compared to triblock copolymers.

Interestingly, the phase image of the SAPD_H20_ triblock copolymer membrane revealed significant chemical heterogeneity. Height and phase images were superimposable and showed corresponding higher regions in the topology image and lighter regions in the phase image, with a phase shift of 7.5 deg, indicating areas with slightly differing hydrophilicity. This observation aligns with the higher CA values for the SAPD_H20_ triblock copolymer membranes, supporting the hypothesis that the underlying “I-shaped” or “U-shaped” conformations of the triblock copolymer chains adsorbed on the hydrophilic substrate creates a chemically inhomogeneous surface on the QCM-D sensor. This inhomogeneity translates to surface patches because less compact and structurally heterogeneous membranes are formed, as indicated by AFM phase imaging. Consequently, while SAPD triblock membranes are overall thicker, their localized variations in thickness contribute to a lower Young’s modulus when probed by AFM. The LB-deposited triblock copolymer membrane displayed a higher phase shift of 13.3 deg, which correlates with its increased roughness and the presence of both “I-” and “U-shaped” polymer conformations (Figure 6A). In comparison, the SAPD_TBS_ membranes showed the most pronounced phase shifts, consistent with their rougher and less regular surface topography, originating from the “salting-out” effect.

**Figure 7:**
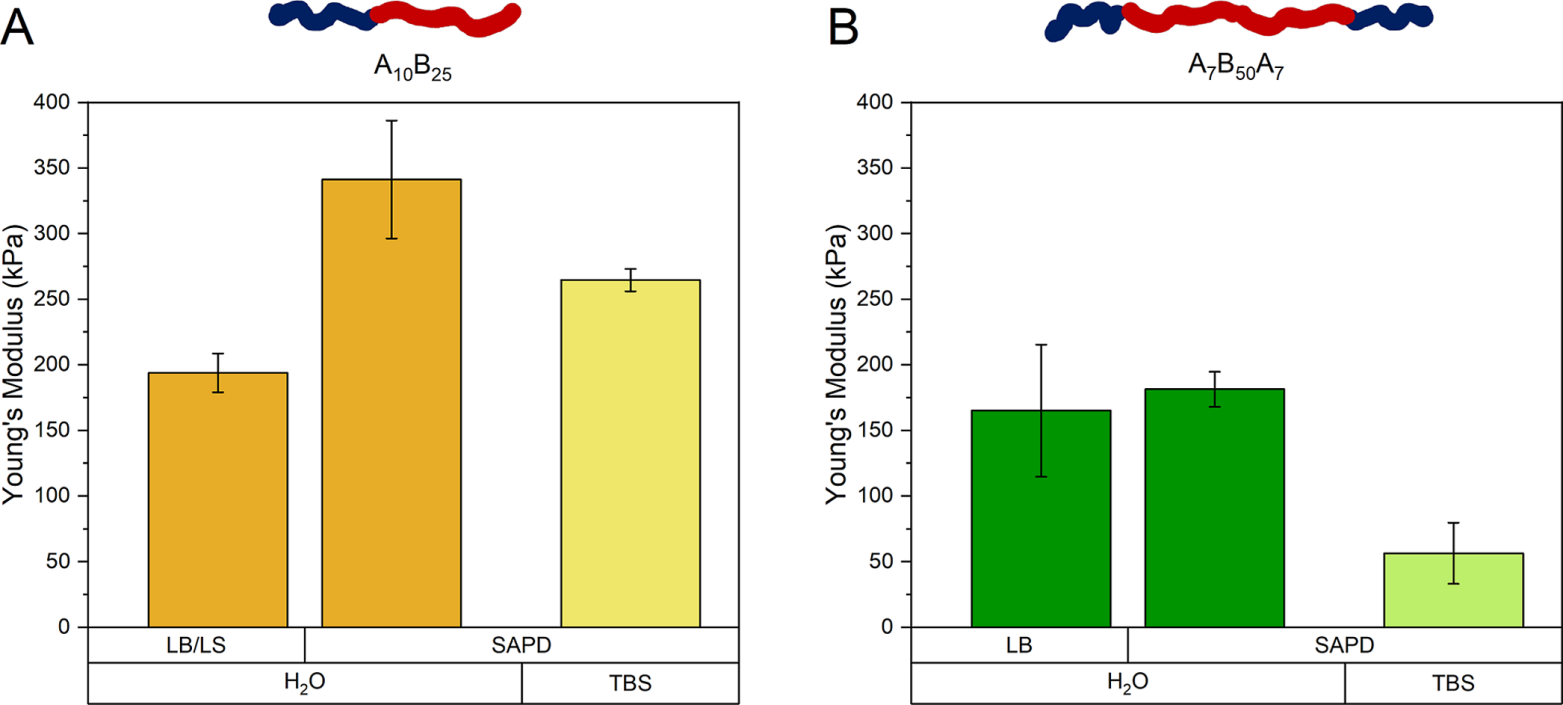
Young’s modulus measured in water for diblock (A) and triblock (B) copolymer membranes prepared using the LB/LS and SAPD methods.

To assess the mechanical properties of the membranes, force spectroscopy was performed. Multiple force curves were analyzed to evaluate the stiffness of diblock and triblock copolymer membranes (Figure 7). This method allowed for the measurement of polymer membrane response to an applied force at the nanoscale level. To this end, the Young’s modulus (*E*) was quantified, representing the ratio of the applied stress to the resulting axial strain within the linear elastic region (reversible deformation, see eq 6).^32^ It is important to note that the force applied to the membranes during this measurement exceeded that used for topology imaging, providing insights into chain interactions upon deformation.

The Young’s modulus of the LB/LS-deposited diblock copolymer membrane (194 ± 15 kPa) was lower than that of the SAPD_H_2_O_ membrane (341 ± 45 kPa). This difference likely originates from the strong entanglement of the PDMS chain ends in the SA-deposited membranes, which increases their resistance to mechanical deformation. The spontaneous self-assembly process in SAPD results in a thermodynamically favorable chain arrangement, producing a denser and more tightly packed membrane, which increases the rigidity and, consequently, the Young’s modulus. In contrast, the molecular organization and packing in the LB/LS method are carefully controlled through the surface pressure during the monolayer formation at the air–water interface. However, this control may be partially lost after the transfer to the solid support due to the loss of surface pressure constraints. It results in higher molecular freedom, chain relaxation and interdigitation of the elongated PDMS chains. This allows for chain sliding during compression, resulting in a less rigid membrane.

In the case of the membranes of triblock copolymers, E values were generally lower, at 165 ± 50 kPa for LB and 181 ± 13 kPa for SAPD_H_2_O_. This decrease is possibly due to their higher molecular weight (with the longer PDMS block leading to increased chain flexibility), higher molecular weight dispersity and the coexistence of mixed “I-” and “U-shaped” chain conformations, which make these membranes less stiff than their diblock counterparts.^55^ Additionally, interdigitation and entanglement of PDMS chains are less common in the triblock copolymer due to the lower conformational freedom of the central PDMS block. This leads to a decrease in membrane stiffness, and minimizes the differences in mechanical properties arising from the preparation method.

In both diblock and triblock membranes, the use of buffer solutions in the SAPD process significantly reduced the Young’s modulus, with values of 265 ± 9 kPa for the diblock and 56 ± 23 kPa for the triblock. This decrease of E values is likely attributed to the “salting-out” effect of the buffer, which disrupted the polymer chain organization, thereby reducing membrane rigidity.

Overall, all E values were lower than previously reported values for polymer membranes, such as 17 ± 11 MPa for polymersomes^33^ or 24 MPa for SSPMs prepared with block copolymers based on PMOXA and PDMS.^7^ Notably, the higher E values in these examples can be attributed to the differences in experimental conditions: the former involved intact vesicles on solid supports, while the latter examined Biotin-functionalized membranes, both of which significantly influence the mechanical properties. Since the membranes investigated in this study are composed of identical materials, the force spectroscopy results highlight the differences in mechanical properties arising from variations in the internal organization of the polymer membranes.

## Conclusions

Solid-supported polymer membranes have been generated by two different deposition methods to determine whether there are differences in their properties and inner morphology. A deep understanding of such molecular aspects bridges the gap between different membranes reported in the field and represents a key approach for promoting membranes for specific applications. By selecting the same amphiphilic copolymers for the generation of the membranes (diblock and triblock, respectively) and performing the LB/LS and SAPD deposition methods in conditions as comparable as possible, we took the advantage to concentrate on the differences resulting from the self-assembly process. Membranes were analyzed by a combination of methods to obtain various membrane properties (surface coverage, thickness, wettability, topographical and mechanical properties), providing insights into their molecular organization.

For the selected diblock copolymer, both LB/LS and SAPD produced homogeneous membranes with comparable structural and surface characteristics. The only observed difference was a slight variation in nanoscale topology and E values originating from the potential interdigitation and entanglement of PDMS blocks. On the contrary, the triblock copolymer membranes showed different properties depending on the deposition method. Triblock membranes prepared via SAPD exhibited increased thickness, reduced wettability, and more pronounced morphological features compared to those prepared by LB methods. These differences stem from the dissimilar conformational freedom of the polymer chains during the self-assembly process that takes place in different environmental conditions (liquid sequences in SAPD compared to air–water interface in LB/LS). In liquid sequences specific for the SAPD process, the polymer chains have a high conformational freedom during the solvent exchange and self-assembly process. Additionally, a small fraction of polymers adsorbed onto the solid substrate, most likely in the “U-shaped” conformation, which may increase membrane thickness by possible superposition. In contrast, LB deposition constrains the polymer conformation at the air–water interface early in the process, promoting a “U-shape” for the triblock copolymer. With further compression, the PDMS block either bends into an inverted “U-shape” with both PMOXA blocks solvated, or move one PMOXA block out of the water, adopting a coiled “I-shape”, which explains the thickness difference. More details regarding the presence of different triblock copolymer conformations would require advanced scattering techniques (e.g., neutron reflectometry) and asymmetrically modified polymer probes (e.g., one partially or completely deuterated PMOXA block). However, these modifications could alter membrane formation and are beyond the scope of this study. Additionally, the comparison of the membranes resulting from the SAPD process in water or TBS indicated a decrease of the membrane thickness due the “salting-out” effect present in buffer, which influences the molecular organization of the polymers. This emphasizes the importance of buffer selection in the experimental design for obtaining membranes with desired properties.

Our study indicates that the two different methods of deposition used for preparation of solid-supported planar membranes are not significantly affecting the properties of diblock copolymer membranes, with exception of the membrane rigidity. On the contrary, in the case of the triblock copolymer, the resulting membranes exhibit significant differences in properties, making the choice of the method dependent on the specific requirements of the desired application. The compatibility between the deposition method and the functional moieties or (bio)molecules to be inserted into the membrane needs to be carefully evaluated, particularly key factors including solvent susceptibility, deposition duration, and postfunctionalization. Our approach and analysis provide a solid basis for selecting the most suitable deposition method to tailor membrane properties for specific applications.

## Supporting Information Available

It contains additional experimental data including surface pressure–area isotherm characteristics, Langmuir monolayer transfer characteristics, additional surface coverage data, additional membrane wettability data, membrane stability data, and microscopic images of deposited membranes (PDF)

## Abbreviations

AFM: atomic force microscopy
BCPs: block copolymers
BSA: bovine serum albumin
CA: contact angle
EtOH: ethanol
LB: Langmuir–Blodgett
LS: Langmuir–Schaefer
MMA: mean molecular area
PDMS: poly(dimethylsiloxane)
PMOXA: poly(2-methyl-2-oxazoline)
QCM-D: quartz crystal microbalance with dissipation monitoring
*R*_q_: RMS roughness
SAPD: solvent-assisted polymer deposition
SP: surface pressure
SSPMs: solid-supported polymer membranes
TBS: Tris-buffered saline
TR: transfer ratio
