# Supplementary material for: Solid-Supported Polymer Membranes: How Different Deposition Methods Influence Their Inner Morphology and Properties
Source: Langmuir. 2025 Apr 23;41(17):10893–907. doi: 10.1021/acs.langmuir.5c00105 (PMC12060281; doi:10.1021/acs.langmuir.5c00105)
Supplement: Supplementary file 1 — la5c00105_si_001.pdf [file la5c00105_si_001.pdf]

Supporting Information

Solid-supported polymer membranes: How different deposition methods influence their inner morphology and properties

Moritz S. Muthwill,[†1,2] *Manuel Kraus,*[†1] *Maryame Bina,*[1] *Mirela Malekovic,*[1] *Ionel Adrian Dinu,*[*1] *and Cornelia G. Palivan*[*1,2]

[1]Biointerfacing nanomaterials Group, Department of Chemistry, University of Basel, Mattenstrasse 22, BPR 1096, 4002, Basel, Switzerland

[2]NCCR Molecular Systems Engineering, Mattenstrasse 22, BPR 1095, 4002, Basel, Switzerland

[†]These authors have contributed equally

Corresponding authors

*Cornelia G. Palivan – E-mail: cornelia.palivan@unibas.ch

*Ionel Adrian Dinu – E-mail: adrian.dinu@unibas.ch

Supporting Data

Surface pressure-area isotherms

Phase behavior of di- and triblock copolymers were analyzed by performing surface pressure - area compression isotherms. Characteristic data for both polymers are depicted in Table S1. Collapse points values correspond to the maximum of the surface pressure before monolayer collapse occurs and their respective mean molecular area at that moment. Limiting molecular areas are determined by extrapolation to zero surface pressure from the linear increase before reaching the collapse point, while the lift-off area describes the area at which the surface pressure deviates from zero.

Table S1: Characteristic data recorded for Langmuir surface pressure – area isotherms of diblock ($A_{10}B_{25}$) and triblock ($A_7B_{50}A_7$) copolymers.

Block copolymer	Collapse point		Limiting molecular area (nm^2)	Lift-off area (nm^2)
	Surface pressure (mN/m)	Mean molecular area (nm^2)		
$A_{10}B_{25}$	50.6 ± 0.9	0.71 ± 0.02	1.69 ± 0.05	6.11
$A_7B_{50}A_7$	51.3 ± 0.5	0.93 ± 0.07	2.56 ± 0.09	10.77

Langmuir monolayer transfer

Characteristic data for Langmuir monolayers transfer methods to produce polymer membranes are depicted in Table S2. Membranes were deposited on silicon wafers (2x2cm) at a constant surface pressure. The deposition time correlates to the size of the sample and the dipping speed. Background loss refers to the reduction in monolayer area due to the block copolymers escaping the trough barriers.

Table S2: Characteristic data recorded for Langmuir monolayer transfer methods (LB and LS) of diblock ($A_{10}B_{25}$) and triblock ($A_7B_{50}A_7$) copolymers.

Block copolymer	Layer	Surface pressure (mN/m)	Deposition time (s)	Background loss (mm^2/s)
$A_{10}B_{25}$	1 (LB)	41.5	2365 ± 25	-0.32 ± 0.04
	2 (LS)		524 ± 13	-0.33 ± 0.04
$A_7B_{50}A_7$	1 (LB)	41.5	2582 ± 80	-0.17 ± 0.05

Effect of sample geometry and handling for BSA surface coverage measurements

BSA measurements for LB/LS samples require deposition on suboptimal substrates (QCM-D sensors) and extensive handling. The effect of these parameters was investigated by subjecting SAPD samples to the same procedure (namely prolonged drying in air and reinsertion into QCM-D flow cell) and measuring the surface coverage with BSA after (Table S3).

Table S3: Surface coverage of diblock ($A_{10}B_{25}$) and triblock ($A_7B_{50}A_7$) copolymer $SAPD_{TBS}$ membranes by BSA adsorption assay in QCM-D.[a]

Block copolymer	Treatment	Δf_{BSA} (Hz)	Surface coverage
$A_{10}B_{25}$	Removed	-6.0 ± 0.2	76 ± 1 %
	Not Removed	-1.9 ± 0.8	95 ± 5%
$A_7B_{50}A_7$	Removed	-3.6 ± 0.1	86 ± 1 %
	Not Removed	-0.9 ± 1.0	96 ± 4%
Control		-25.3 ± 2.0	0 ± 8 %

[a]Membranes were deposited on SiO2-functionalised QCM-D sensors. As a control, a bare sensor was used. Samples were either measured directly after deposition without removal from the flow cell, or removed, allowed to dry in air and reinserted before the BSA measurement. Values were averaged from three replicates and are indicated with the standard deviation.

Influence of substrate on membrane wettability

Since for monitoring of SAPD by QCM-D, SiO_2-functionalised sensors were used, whereas silicon wafers with usually lower roughness were used for SAPD comparison with LB/LS, the influence of the substrate used for SAPD was investigated by measuring the CA directly after deposition (day 0) and after 7 days storage in air (day 7) (Figure S1). No significant impact of the substrate on membrane wettability was observed.

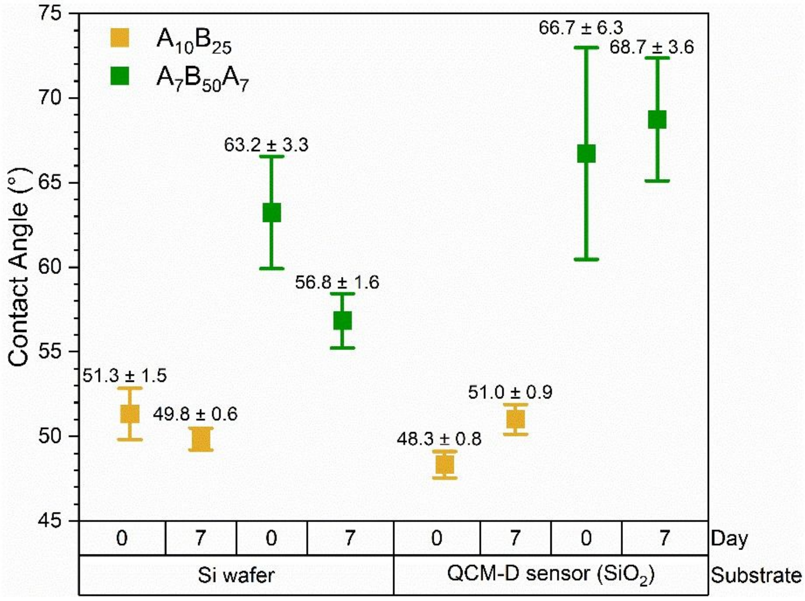

Figure S1: Influence of substrate on wettability of diblock ($A_{10}B_{25}$) and triblock ($A_7B_{50}A_7$) copolymer membranes deposited by SAPD measured by water contact angle.

Microscopic characterisation of polymer membranes

Brightfield microscopy images of deposited membranes were recorded during ellipsometry measurement in air (Figure S2). They give first indications of defect density and inhomogeneity within the membranes, which are generally low. All membranes exhibit dark spots compared to the surrounding area, indicating the presence of locally inhomogeneous topology. Additionally, an increased density of such spots can be observed for LB/LS membranes, likely due to the open nature of the deposition method.

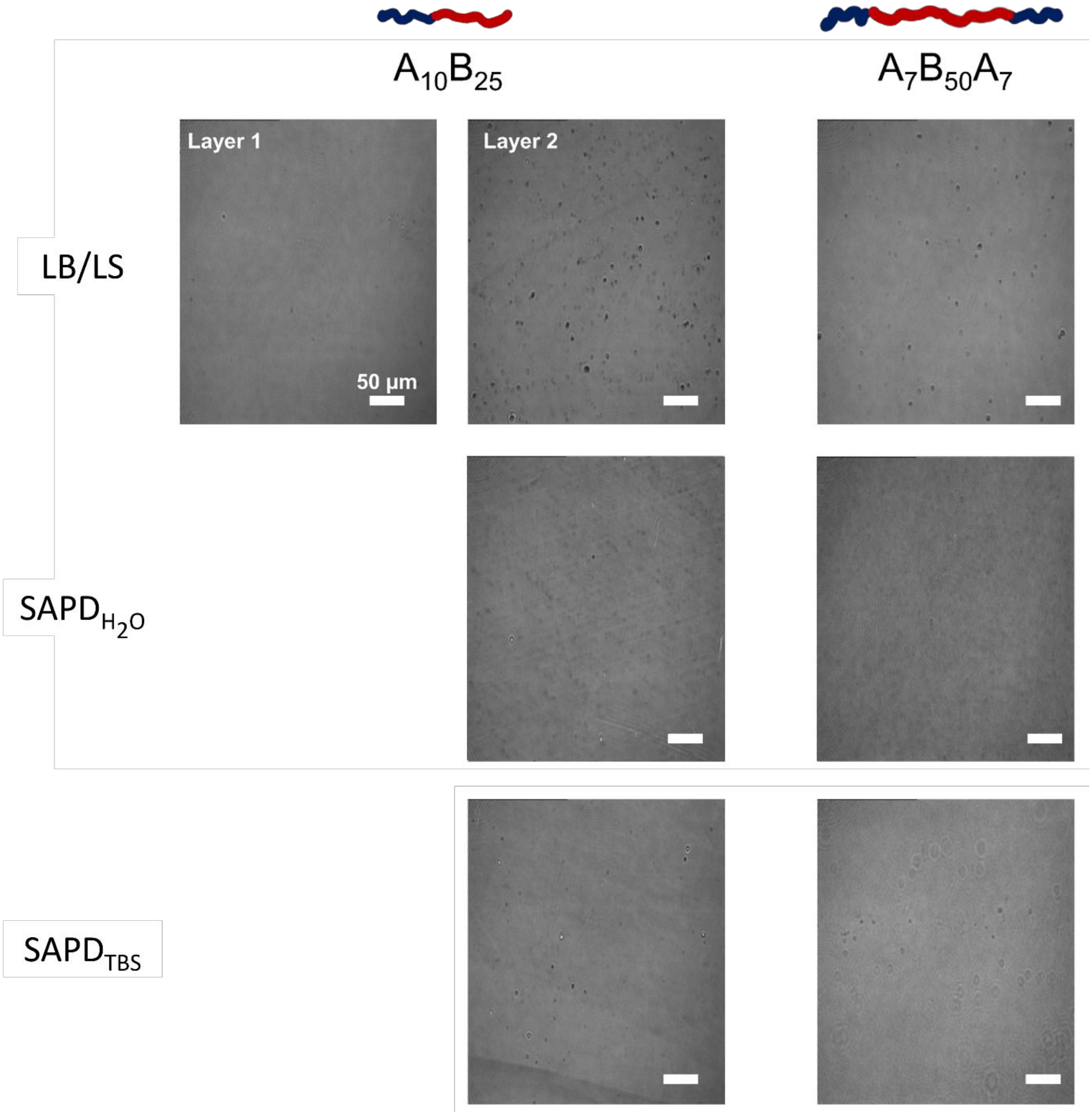

Figure S2: Exemplary microscopic images of diblock ($A_{10}B_{25}$) and triblock ($A_7B_{50}A_7$) copolymer membranes deposited by the LB/LS technique and the SAPD method.

Stability of deposited membranes over time

Membrane stability was assessed by storing deposited membranes in air and darkness for 7 days (Figure S3). Changes in membrane thickness (measured by ellipsometry) and surface properties (determined by contact angle measurements) were recorded and compared to values obtained on the day of deposition.

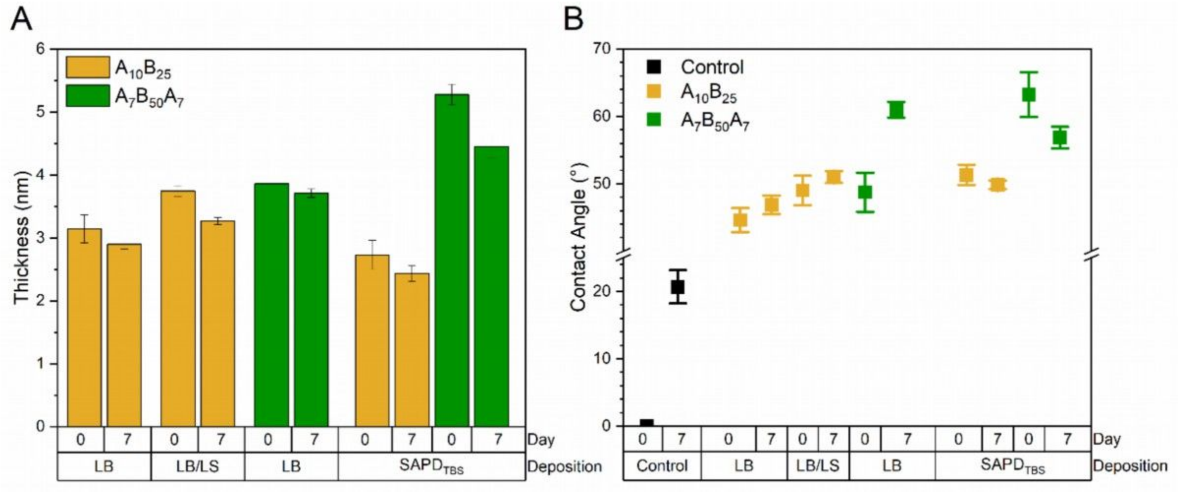

Figure S3: Stability of diblock ($A_{10}B_{25}$) and triblock ($A_7B_{50}A_7$) copolymer membranes deposited by LB(/LS) and SAPD$_{TBS}$ on Si wafers. Stability is investigated by thickness (A) and contact angle measurements (B) directly after deposition and after subsequent storage in air for 7 days. As control, a bare and hydrophilized Si wafer was used.

Peak-to-valley distances

Characteristic data for vertical and horizontal peak-to-valley distances for deposited membrane topologies are listed in Table S4. For the vertical distance, five squares of equal dimensions (300x300 nm) were analyzed in each membrane sample within the AFM software. These values correspond to the average vertical distance between highest to lowest point and are an indication of the membrane homogeneity and height distribution. For the horizontal distance, height profiles of cross sections across the squares were analyzed and the horizontal distance between highest peak and lowest valley was determined. Indicated values correspond to the average and standard deviation.

Table S4: Characteristic vertical and horizontal peak-to-valley distances of diblock ($A_{10}B_{25}$) and triblock ($A_7B_{50}A_7$) copolymer membranes as measured by AFM height images.

Distance orientation	Block copolymer	Deposition Method		
		LB / LS	$SAPD_{H2O}$	$SAPD_{TBS}$
Vertical (z-direction)	$A_{10}B_{25}$	0.9 ± 0.1 nm	1.2 ± 0.1 nm	6.1 ± 0.5 nm
	$A_7B_{50}A_7$	1.3 ± 0.1 nm	1.6 ± 0.1 nm	9.2 ± 1.5 nm
Horizontal (xy-direction)	$A_{10}B_{25}$	28.6 ± 4.3 nm	150.1 ± 12.7 nm	169.7 ± 14.1 nm
	$A_7B_{50}A_7$	53.8 ± 7.6 nm	191.9 ± 18.7 nm	209.2 ± 33.0 nm
